
JOURNAL INFORMATION
==============================
NLM Title Abbreviation: ScientificWorldJournal
Journal ID: ScientificWorldJournal
NLM Journal ID: 101131163
Journal ID: TSWJ

The Scientific World Journal

ISSN: 2356-6140
EISSN: 1537-744X
Publisher: Wiley

ARTICLE INFORMATION
==============================
PMCID: PMC5901315
DOI: 10.1100/tsw.2007.170
Article ID: 915364
Article version: 1
Subjects: Publisher's Note

Publication of a Special Issue: Gene Dosage Effect During Embryonic Development

Redhead Claire
Publication date: 2007
Electronic publication date: 2007 Jun 12
Volume: 7
First page: 170
Last page: 170
Copyright: Copyright © 2007 Claire Redhead.
Copyright year: 2007
License: This is an open access article distributed under the Creative Commons Attribution License, which permits unrestricted use, distribution, and reproduction in any medium, provided the original work is properly cited.
License URL: https://creativecommons.org/licenses/by/3.0/

==============================
Publisher's Note
*TheScientificWorldJOURNAL* (2007) 7, 1007
TSW Development & Embryology
ISSN 1749-4958; DOI 10.1100/tswde.2007.2

TheScientificWorldJOURNAL
www.thescientificworld.com

### Publication of a Special Issue: Gene Dosage Effect During Embryonic Development

Claire Redhead
*TheScientificWorld*

**E-mail:** credhead@thescientificworld.com

*Published June 12, 2007*

---

TheScientificWorldJOURNAL and TSW Development & Embryology are pleased to have published the special issue 'Gene Dosage Effect During Embryonic Development', a collection of peer-reviewed papers on a range of key topics in this field. The issue has been organized by Dr. Yann Hérault (Group Leader, Molecular Embryology and Morphogenesis, CNRS, France) and specific topics covered are:

- The 22q11.2 Deletion Syndrome: A gene dosage perspective
- DYRK1A (dual-specificity tyrosine-phosphorylated and regulated kinase 1A): a gene with dosage effect during development and neurogenesis
- Developmental defects in trisomy 21 and mouse models
- The William Beuren Syndrome
- Transcriptional consequences of gene dosage imbalance on cerebellum development

The Publisher wishes to thank the authors and reviewers, and especially acknowledges the contribution of Dr. Hérault in bringing about the publication of this special issue.

**This article should be cited as follows:**

Redhead, C. (2007) Publication of a special issue: gene dosage effect during embryonic development. *TSW Development & Embryology*, **2**, 72. DOI 10.1100/tswde.2007.2.

*Corresponding author.
©2007 with author.
